# Early clinical indicators of acute kidney injury caused by administering high-dose methotrexate therapy to juvenile pigs

**DOI:** 10.3389/fneph.2023.1193494

**Published:** 2023-09-12

**Authors:** Randal K. Buddington, Thomas Wong, Karyl K. Buddington, Torben S. Mikkelsen, Xueyuan Cao, Scott C. Howard

**Affiliations:** ^1^ College of Health Sciences, University of Memphis, Memphis, TN, United States; ^2^ Division of Endocrinology, University of Tennessee Health Sciences Center (UTHSC), Memphis, TN, United States; ^3^ Department of Biological Sciences, University of Memphis, Memphis, TN, United States; ^4^ Department of Clinical Medicine, Aarhus University Hospital, Aarhus, Denmark; ^5^ College of Nursing, Resonance, Memphis, TN, United States; ^6^ Department of Health Promotion and Disease Prevention, University of Tennessee Health Science Center, Memphis, TN, United States

**Keywords:** methotrexate, creatinine, acute kidney injury, chemotherapy, pig, animal model, clearance

## Abstract

**Introduction:**

Early identification of compromised renal clearance caused by high-dose methotrexate (HDMTX) is essential for initiating timely interventions that can reduce acute kidney injury and MTX-induced systemic toxicity.

**Methods:**

We induced acute kidney injury (AKI) by infusing 42 juvenile pigs with 4 g/kg (80 g/m2) of MTX over 4 hours without high-volume alkalinizing hydration therapy. Concentrations of serum creatinine and MTX were measured at 15 time points up to 148 hours, with 10 samples collected during the first 24 hours after the start of the HDMTX infusion.

**Results:**

During the first 28 hours, 81% of the pigs had increases in the concentrations of serum creatinine in one or more samples indicative of AKI (i.e., > 0.3g/dL increase). A rate of plasma MTX clearance of less than 90% during the initial 4 hours after the HDMTX infusion and a total serum creatinine increase at 6 and 8 hours after starting the infusion greater than 0.3 g/dL were predictive of AKI at 28 hours (*p* < 0.05 and *p* < 0.001, respectively). At conclusion of the infusion, pigs with a creatinine concentration more than 0.3 g/dL higher than baseline or serum MTX greater than 5,000 μmol/L had an increased risk of severe AKI.

**Conclusions:**

Our findings suggest that serum samples collected at conclusion and shortly after HDMTX infusion can be used to predict impending AKI. The pig model can be used to identify biological, environmental, and iatrogenic risk factors for HDMTX-induced AKI and to evaluate interventions to preserve renal functions, minimize acute kidney injury, and reduce systemic toxicity.

## 1 Introduction

High-dose methotrexate (HDMTX), defined as doses of 1 g/m^2^ of body surface area and higher, is used to treat a variety of cancers, including acute lymphoblastic leukemia, non-Hodgkin lymphoma, and osteosarcoma (1). Supportive care during and after HDMTX is critical to reduce the risk of toxicity and especially acute kidney injury (AKI). Methotrexate (MTX)-induced AKI is thought to be caused when MTX crystalizes in the renal tubular lumen, leading to tubular toxicity (1–3). Because low urine pH decreases the solubility of MTX and the metabolite 7-hydroxy-methotrexate (7OH-MTX) and increases MTX uptake by renal tubular epithelial cells, hyperhydration and urine alkalinization are used to ensure a urine pH ≥ 7.0 until the serum MTX level is < 0.2 µM to reduce both crystal-induced injury and cellular damage after internalization and polyglutamation within urothelial cells (1, 4, 5).

In children treated with HDMTX, the incidence of AKI (1.5-fold increase in plasma creatinine concentration) is seen in up to 40% of all children receiving 24-hour infusions of HDMTX at 8 g/m² (6). Most children recover from HDMTX-induced AKI (7), but renal functions can be compromised for weeks (8) and cause delays in subsequent cancer treatment. Early detection of nephrotoxicity for immediate initiation of effective interventions is essential to avoid irreversible damage and permanent nephron destruction. Currently, an increase in serum creatinine concentration or a persistently high level of serum MTX after the HDMTX infusion are the most commonly used indicators of impaired renal function.

Serum creatinine remains a widely used biomarker for AKI (9, 10). However, creatinine has limitations as an indicator of renal clearance and injury (11–13) and significant increases in concentration may not occur for 24 hours or longer after the onset of renal injury. Alternative markers of renal injury (14–18) suffer the same shortcomings as creatinine, in that appearance in the serum or urine occurs after renal injury has already occurred.

The impact of early detection of MTX-induced AKI and the efficacy of early interventions have not been methodically studied, in part because no relevant large animal model has been developed in which MTX-induced impaired renal clearance can be reliably produced. This includes determining if early treatment with glucarpidase, an enzyme that converts MTX into non-toxic metabolites and is currently used as a rescue drug in cases with severely delayed MTX elimination and AKI (7, 19, 20), will prevent the further deterioration of renal functions.

Rodent models using mostly mice and rats have dominated HDMTX research. Despite the insights gained into the mechanisms of toxicity, differences in renal characteristics and the small size of rodent models limit the translation of findings into clinical care. Non-human primates share similarities with humans, but high costs, and specialized care and caging requirements limit their use for preclinical studies. This study used juvenile pigs as a translational large animal model that is relevant for patients receiving HDMTX. Pigs have a renal structure and renal functions that are similar to humans, as evident from recent pig-to-human kidney transplants. Pigs are sufficiently large to allow for central venous access and serial blood sampling, and reliably develop AKI compatible with the HDMTX-induced renal toxicity in humans (as shown in the present study). Furthermore, pigs are suitable for replicating the chemotherapy and supportive care procedures used in patients and for evaluating interventions. Pigs have been used to evaluate the pharmacokinetics of low-dose MTX (21), as a preclinical model to assess the safety of MTX administration directly into the fourth ventricle (22), and to study the relationship between renal clearance and glomerular filtration (23). Moreover, porcine models for gastrointestinal damage associated with the chemotherapeutic agent doxorubicin replicate the conditions in humans (24, 25).

## 2 Materials and methods

### 2.1 Pigs and their care

All aspects of the research involving animals were approved by the University of Memphis Institutional Animal Care and Use Committee (protocol #0745). A total of 56 weaned, specific pathogen-free male pigs of a consistent genetic lineage were obtained at approximately 20 days of age from a commercial source. Male pigs were used because of the possible increased risk of nephrotoxicity caused by HDMTX (26). The pigs were initially group housed in a Polydome Litter Saver Pig Nursery with environmental enrichment during a 3- to 5-day period of acclimation to laboratory settings, the experimental diet, and human contact. The room was maintained at 22°C ± 1°C, with a 12 : 12 hour light : dark cycle. The pigs were weighed daily and had frequent human interactions to facilitate chemotherapy administration, handling, and sampling.

To mimic nutrition support for cancer patients and reduce the magnitude of weight loss, the pigs were fed a premium dog food (Purina Pro Plan®) to increase the protein and energy supply relative to standard pig feed. Prior to dosing, a catheter (3.5 French single-lumen Umbili-Cath™; Utah Medical Products, Inc., Midvale, UT, USA) was surgically placed in the right jugular vein, then tunneled to, and exteriorized on, the dorsal surface at the base of the neck, and secured using a Statlock® Foley Stabilization Device (Bard Medical, Covington, GA, USA). The catheter was used for administration of MTX, the antiemetic ondansetron [Pfizer, Inc., NY, USA; 0.15 mg/kg, IV (intravenously)], and leucovorin (0.5 mg/kg, IV), and for the collection of blood samples. For the surgery, the pigs were fasted overnight and sedated [Telazol®; Fort Dodge Animal Health, Fort Dodge, IA, USA; 3–4 mg/kg, IM (intramuscularly)]. A sterile field was prepared, and anesthesia was induced and maintained using isoflurane (1%–5% with oxygen). The pigs received postoperative analgesia immediately after surgery [Carprofen®; Fort Dodge Animal Health, Fort Dodge, IA, USA; 2-4 mg/kg, IM and after 24 hours [Metacam®; Boehringer Ingelheim Animal Health, Duluth, GA, USA; 0.2 mg/kg, per os (orally)]. The pigs were individually housed after the surgery. A prophylactic antibiotic (cefazolin; Bayer Animal Health; 10 mg/kg, IV) was given at the conclusion of the surgery and for 2 days post surgery. The catheters were flushed 2 or 3 times daily with heparinized saline (50 units/mL).

### 2.2 Administration of MTX

The pigs were allowed to recover from surgery and were feeding and gaining weight before starting MTX dosing. After collection of a baseline blood sample and administration of the antiemetic ondansetron (0.15 mg/kg; IV), the dose of MTX was infused over a 4-hour period (Abbott Plus XL3) during which the pigs were awake and resting in slings. To reduce the risk of gastrointestinal and hematologic toxicities, leucovorin (0.5 mg.kg, IV) was provided to all pigs *via* the jugular catheter at hours 28 and 52 after the start of the MTX infusion.

The dose of MTX necessary for a single dose to induce renal toxicity was determined using 14 pigs and by escalating the dose from 0.4 g/kg (*n* = 2), which is equivalent to a human dose of 8 g/m^2^, to 1.2 g/kg (*n* = 4) and then to 2 g/kg (*n* = 8), which still did not cause an increase in serum creatinine concentration within 48 hours (data not reported). Escalation of the MTX dose to 4 g/kg (equivalent to 80 g/m²) induced renal toxicity based on increased serum creatinine concentration and delayed clearance of MTX. The MTX solutions were prepared by solubilizing MTX powder of the desired concentration (2.4, 4, or 8 g/L) in 800 mL of deionized water while stirring and slowly adding 10% sodium hydroxide (NaOH) until most of the MTX was dissolved (solution pH was 7–9). To dissolve the remaining MTX, a 1% NaOH solution was gradually added together with 2.6 g of NaCl and 9 g of benzyl alcohol while continuously stirring until all components were dissolved. The final pH was adjusted to 7.4 using 1% hydrochloric acid (HCl) and the volume was increased to 1 L using deionized water. The prepared MTX solution was placed in intravenous bags and infused using IV sets with a 0.22-micron inline filter at a rate of 12.5 mL/kg/h (250 mL/m^2^/h) for 4 hours. This infusion rate for 4 hours exceeds the hyperhydration of most pediatric protocols, which recommend 125 mL/m²/h during the HDMTX infusion (1). Unlike in pediatric protocols, additional IV hydration was not provided to the pigs before or after the MTX infusion. The volume of water the pigs were allowed to drink after the MTX infusion was limited to 5 mL/kg every hour for 12 hours. Thereafter water was available *ad libitum*. Urine pH was recorded by pH meter before and after administration of the MTX.

A second dose of MTX was provided to 10 pigs at hour 76 after the start of the first HDMTX infusion. These pigs included seven without evidence of AKI and one each with mild, moderate, and severe AKI at 28 hours after the start of the first dose of MTX.

### 2.3 Assessment of responses to MTX

The pigs were observed a minimum of five times each day to monitor general health (eating, drinking, activity, responsiveness to care staff, and vomiting), and stool consistency (using a Likert scale from 0 for normal to 3 for severe diarrhea). Body weight was recorded daily.

Blood samples (1 mL) were collected in serum separator tubes immediately before (hour 0) and at conclusion of the MTX administration (hour 4) and at hours 5, 6, 8, 10, 12, 14, 16, 22, and 28. Thereafter blood samples were collected daily until day 6 (hour 148) after start of the MTX infusion. The serum was isolated and used for measurement of MTX and creatinine (Vet Axcel^®^ Chemistry Analyzer; Alfa Wassermann). The diagnosis of AKI was defined as a ≥ 0.3 mg/dL increase in serum creatinine concentration relative to the start of the infusion (12). The severity of AKI was characterized as mild, moderate, and severe based on creatinine concentration increases above baseline of 0.3–0.5 (mild), 0.5–1.0 (moderate), and > 1.0 mg.dL (severe).

Serum MTX was measured using an enzyme-based method that was adapted for a microplate assay (27). Briefly, 20 μL of serum (with and without serial dilution in water) was added to 130 μL of human dihydrofolate reductase solution (DHFR; R&D Systems, Inc., Minneapolis, MN, USA; two units in 0.05 M Tris buffer pH 7.5) and incubated for 1 minute at 37°C in the plate reader (Synergy 2; BioTek, Winooski, VT, USA) before adding 50 μL of a mixture of dihydrofolate (Sigma Aldrich, St Louis, MO; 50 mg in 15 mL of 0.5M Tris buffer pH 7.5, with 20% 2-mercaptoethanol) and nicotinamide adenine dinucleotide phosphate (NADPH; Sigma Aldrich, St. Louis, MO, USA; 50 mg in 10 mL of 0.5M Tris buffer pH 7.5). The reaction mixture was incubated for 60 minutes at 37°C, during which the change in absorbance at 340 nm was recorded every 30 seconds. A standard curve was prepared for the initial rate of decline in absorbance in the presence of MTX concentrations ranging from 0 to 20 μmol/L.

The majority of pigs (*n* = 31) were euthanized at hour 148 (Euthasol®; Virbac Animal Health, Fort Worth, TX, USA; 1 mL/4.5 kg). It was necessary to humanely euthanize eight pigs before 148 hours because they had become moribund (unresponsive, weight loss greater than 20%) or had an exceedingly high serum concentration of creatinine (> 7 mg/dL). Another three pigs died suddenly between 24 and 72 hours. The kidneys were removed from all pigs and were inspected externally and internally for gross signs of injury and for visual accumulation of MTX crystals. Other organs were visually inspected.

### 2.4 Statistical analysis

Values presented in tables are means and standard deviations. The rate of decline in the levels of serum MTX (CSC) was defined as change between the MTX concentration at a specific time point and the MTX concentration at the end of the MTX infusion:


(1) CSC=100×(end of infusion serum MTX–serum MTX at time point x)/(end of infusion serum MTX)

Higher CSC values represent faster rates of MTX elimination from the blood. A generalized linear model with mixed effects was used to model CSC values with AKI at 24 hours for the pigs that received the highest MTX infusion. A *p*-value less than 0.05 was considered statistically significant.

## 3 Results

**Figure 1:**
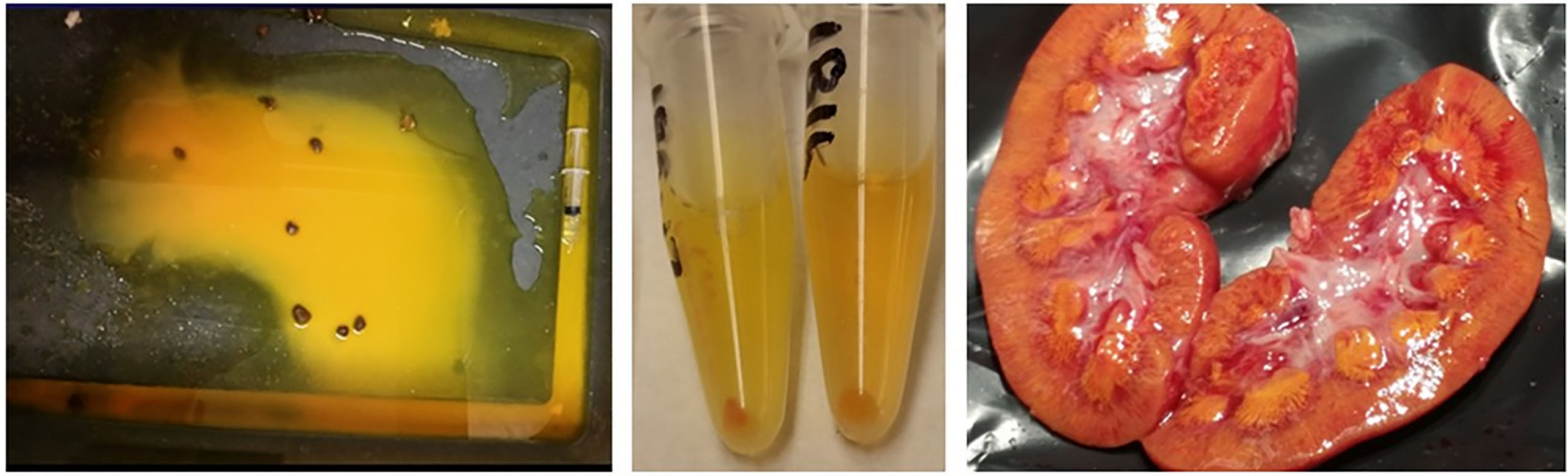
Macroscopic methotrexate crystals were evident in the pen (left panel) and sedimented in the urine (middle panel) during the infusion and in the kidneys (right panel) of animals with acute kidney injury after administering 4 g methotrexate/kg body weight.

The 42 pigs treated with the highest dose of MTX (4 g/kg) survived the infusion, but 11 pigs died or were euthanized before day 6 after the infusion. The high volume of fluid infused with the MTX resulted in high urine output during the MTX infusion. The pH of urine collected prior to and immediately after the MTX infusion averaged 5.8 (± 0.61; range 4.9–7.1), which is similar to previously published pH measurements of pig urine (28). The presence of MTX crystals in the urine during and after administration of the MTX and in the kidneys of pigs that were necropsied at or before hour 28 (**Figure 1**) indicates that the concentration of MTX in the urine exceeded the solubility limit. Diarrhea and vomiting were observed for only a few animals during the first 24 hours after the MTX infusion and for no pigs after 24 hours. The feces collected during the first 24 hours after the MTX infusion had a yellow tinge. An analysis of stool samples revealed the presence of MTX, although concentrations were not determined. Soon after the start of the MTX infusion, the skin of the pigs developed a yellowish appearance, which gradually disappeared during the next 24 hours. Most pigs that received the 4 g/kg of MTX were lethargic for 6 to 12 hours after the infusion, but readily drank the restricted volume of water. Intrarenal accumulation of MTX crystals was seen in the kidneys of all pigs that died or were euthanized within 52 hours after the MTX infusion (**Figure 1**), but not in kidneys from the pigs that were necropsied at 148 hours.

### 3.1 Body weight gain before and after administration of 4 g/kg of MTX

Most of the pigs (92%) gained weight during the 3 days prior to MTX administration (**Table 1**). The average gain of 2.8% of body weight per day (+ 1.8%) is lower than in production settings but was not unexpected, as the pigs had just been weaned, were recovering from surgery, and were isolated after surgery. During the 3 days after the MTX infusion, 30 pigs (70%) lost weight (–0.90%/day ± 1.98%). This is similar to the growth depression of rabbits after high doses of methotrexate (29). The majority of the pigs (72%) began to gain weight again from days 3 to 6 after the MTX infusion (2.28%/d + 3.26%).

**Table 1:** Body weights (kg) of experimental pigs used to evaluate responses to administration of 4 g/kg methotrexate.

Days relative to administration of methotrexate 4 g/kg body weight	Body weight (kg), mean ± SD
–3	5.89 ± 1.68
0	6.33 ± 1.86
+3	6.02 ± 1.62
+6	5.93 ± 1.59

### 3.2 Serum creatinine and incidence of acute kidney injury

**Figure 2:**
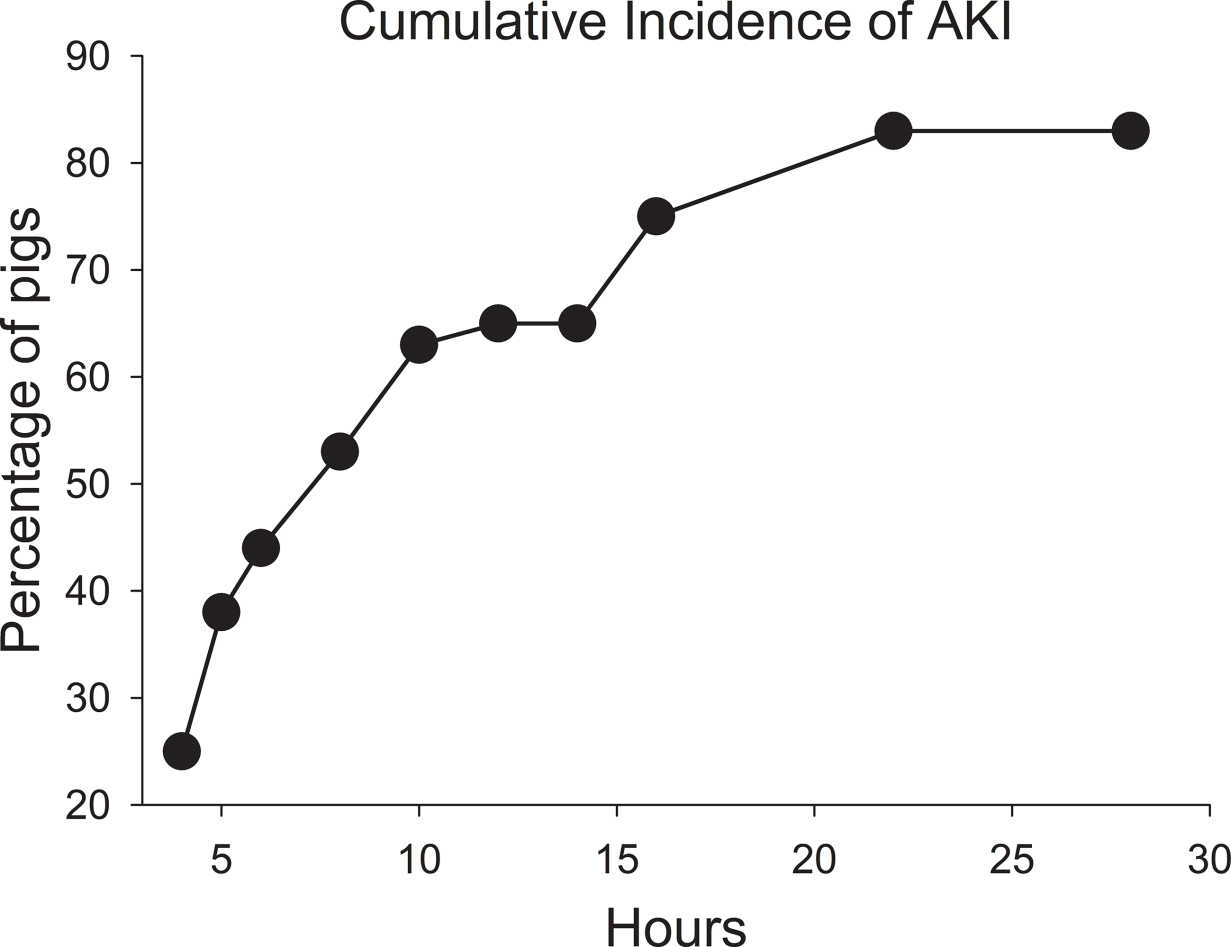
Cumulative incidence of acute kidney injury after starting the infusion of high-dose methotrexate at 4 g/kg body weight.

None of the pigs at the start of the MTX infusion had an elevated serum creatinine concentration indicative of AKI. The percentage of pigs that developed AKI increased after the MTX infusion (**Figure 2**). By 6 hours, nearly 40% of the pigs had an elevated creatinine concentration indicative of AKI. The percentage of pigs with one or more incidences of AKI increased to 70% at 16 hours and 76% at 28 hours, with 81% of the pigs eventually developing AKI of varying severity and duration. Eight pigs (19%) never developed AKI, including three that received the second dose of MTX at 76 hours.

**Figure 3:**
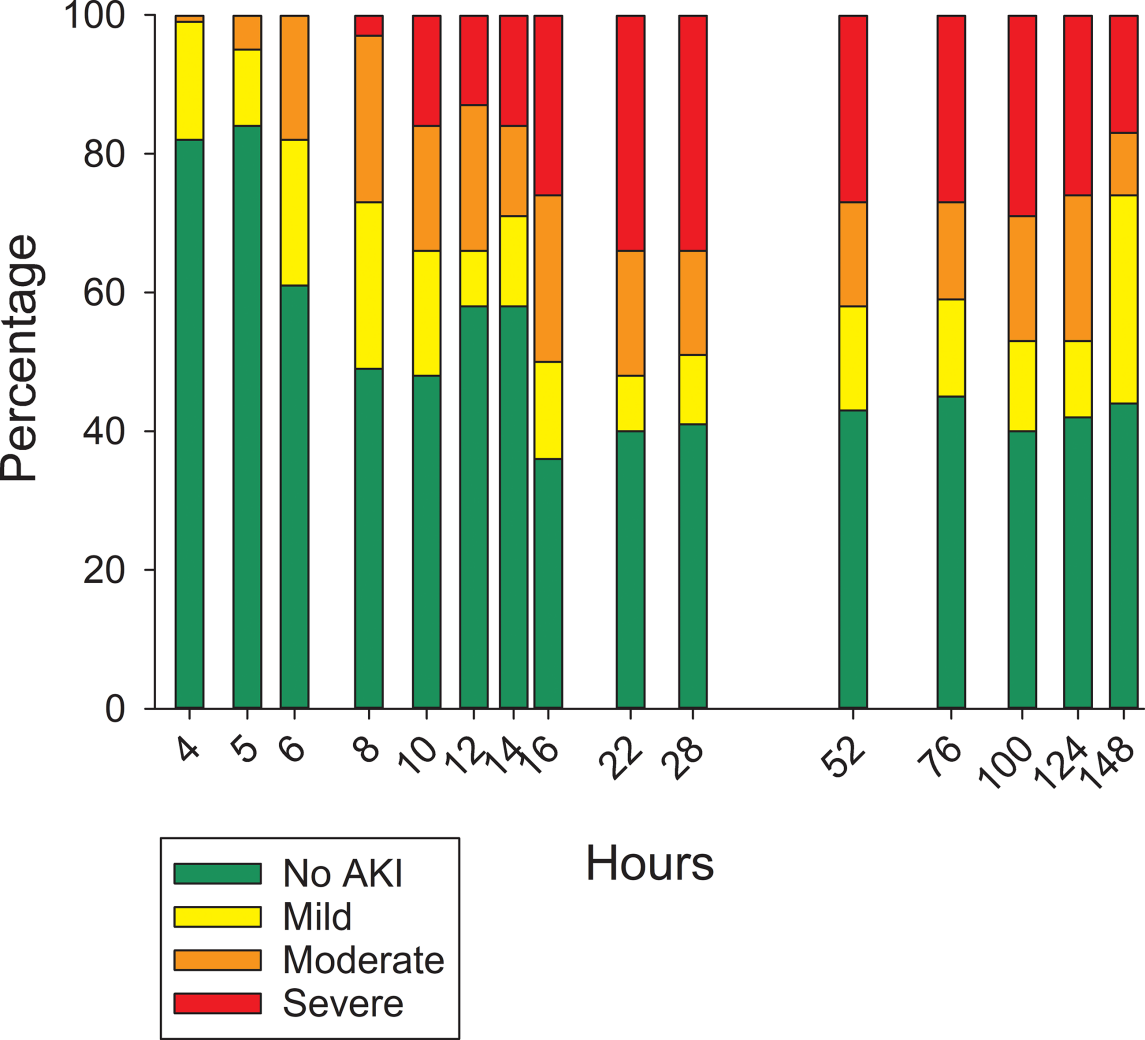
Percentage of pigs with varying severity of acute kidney injury at different time points after initiating the 4-hour infusion of methotrexate at 4 g/kg body weight.

On a population basis, the mean serum creatinine concentration at the end of the 4-hour MTX infusion was unchanged compared with the mean pre-infusion concentration (**Table 2**). Because of the high volume of fluid that was infused (250 mL/m^2^/h) to administer the dose of MTX, at conclusion of the infusion serum creatinine concentration had decreased in 14 pigs (32%). Of these, only four had evidence of AKI at 28 hours. Despite the large fluid volume infused with the HDMTX, eight pigs (19%) had evidence of AKI at conclusion of the infusion, with an average creatinine concentration increase of 0.41 mg/dL (±0.13 mg/dL). Five of these eight pigs developed severe AKI by 28 hours and only two survived to complete the study but with persistent severe AKI. The remaining three pigs developed moderate AKI and two had recovered before hour 148. Among the 26 pigs that developed AKI after hour 4 and conclusion of the first MTX infusion, at 28 hours eight had recovered from earlier evidence of AKI at one or more sampling times and 14 pigs had AKI of similar or worsening severity. The remaining four pigs developed AKI of mild severity after 28 hours (**Figure 3**).

**Table 2:** Serum creatinine concentrations before and after the 4-hour infusion of methotrexate at 4 g/kg with the corresponding relative increases.

Time (hours)	Serum creatinine (mg/dL)	Increase in serum creatinine (mg/dL), mean ± SD
0	0.82 ± 0.19	–
4	0.88 ± 0.23	–0.32 ± –0.15
5	1.00 ± 0.21	0.09 ± 0.24
6	0.96 ± 0.30	0.10 ± 0.23
8	1.13 ± 0.34	0.25 ± 0.23
10	1.17 ± 0.43	0.30 ± 0.34
12	1.29 ± 0.47	0.43 ± 0.42
14	1.22 ± 0.67	0.35 ± 0.57
16	1.51 ± 0.76	0.65 ± 0.69
22	1.72 ± 1.04	0.84 ± 1.00
28	1.82 ± 1.23	0.95 ± 1.18
52	2.08 ± 1.73	1.21 ± 1.71
76	2.30 ± 2.45 (2.71 ± 2.77)	1.45 ± 2.45 (1.90 ± 2.75)
100	2.15 ± 2.33 (2.42 ± 2.68)	1.30 ± 2.31 (1.51 ± 2.65)
124	1.90 ± 2.08 (2.10 ± 2.37)	1.05 ± 2.07 (1.29 ± 2.366)
148	1.34 ± 1.11 (1.29 ± 1.24)	0.49 ± 1.03 (0.49 ± 1.17)

Values in parentheses are for the 10 pigs that received a second dose of methotrexate.

Of the seven pigs with normal serum creatinine concentrations before starting the second dose of MTX at 76 hours, three developed AKI of moderate severity. The two pigs with mild and moderate AKI at the time of the second dose developed moderate and severe AKI, respectively. The pig with severe AKI at the time of the second dose continued to have severely compromised clearance until 124 hours, when it had moderate AKI, and by 148 hours it no longer had AKI based on serum creatinine concentration.

The percentage increases in serum creatinine concentration from baseline were calculated at 16, 28, and 52 hours after the start of the MTX infusion (**Table 3**). There was a 90%–120% increase in serum creatinine concentration for pigs with moderate AKI, defined as a 0.5 - 1.0 mg/dL increase in serum creatinine. Among the pigs with evidence of AKI at 16 hours, those with an increase in creatinine concentration of 50% or less compared with baseline did not have a further increase at 28 and 52 hours. Pigs with more than twofold increase in creatinine concentration at 16 hours had further increases in creatinine concentration at 28 hours. Pigs with severe AKI at 16 hours experienced even higher percentage increases at 28 hours and again at 52 hours, indicating that the severity of AKI continued to increase. This is consistent with the deaths of the pigs that had severe AKI at 16 hours.

**Table 3:** Ratios for the increase in serum creatinine at 16, 28 and 52 hours compared with baseline for pigs with varying severities of acute kidney injury at 12 hours.

Serum creatinine increase and AKI severity at 12 hours	Ratio at 16 hours (%), mean ± SD	Ratio at 28 hours (%), mean ± SD	Ratio at 52 hours (%), mean ± SD
< 0.3 (no AKI)	1.04 ± 0.25 (31)	1.02 ± 0.38 (43)	1.01 ± 0.20 (42)
0.3—0.5 (mild AKI)	1.35 ± 0.38 (18)	1.38 ± 0.40 (15)	1.49 ± 0.19 (16)
0.5—1.0 (moderate AKI)	2.20 ± 0.64 (26)	1.89 ± 0.94 (13)	2.11 ± 0.07 (8)
> 1.0 (severe AKI)	2.78 ± 0.63 (26)	3.89 ± 1.00 (38)	4.69 ± 1.66 (34)

Values in parentheses are the percentages of pigs.

**Figure 4:**
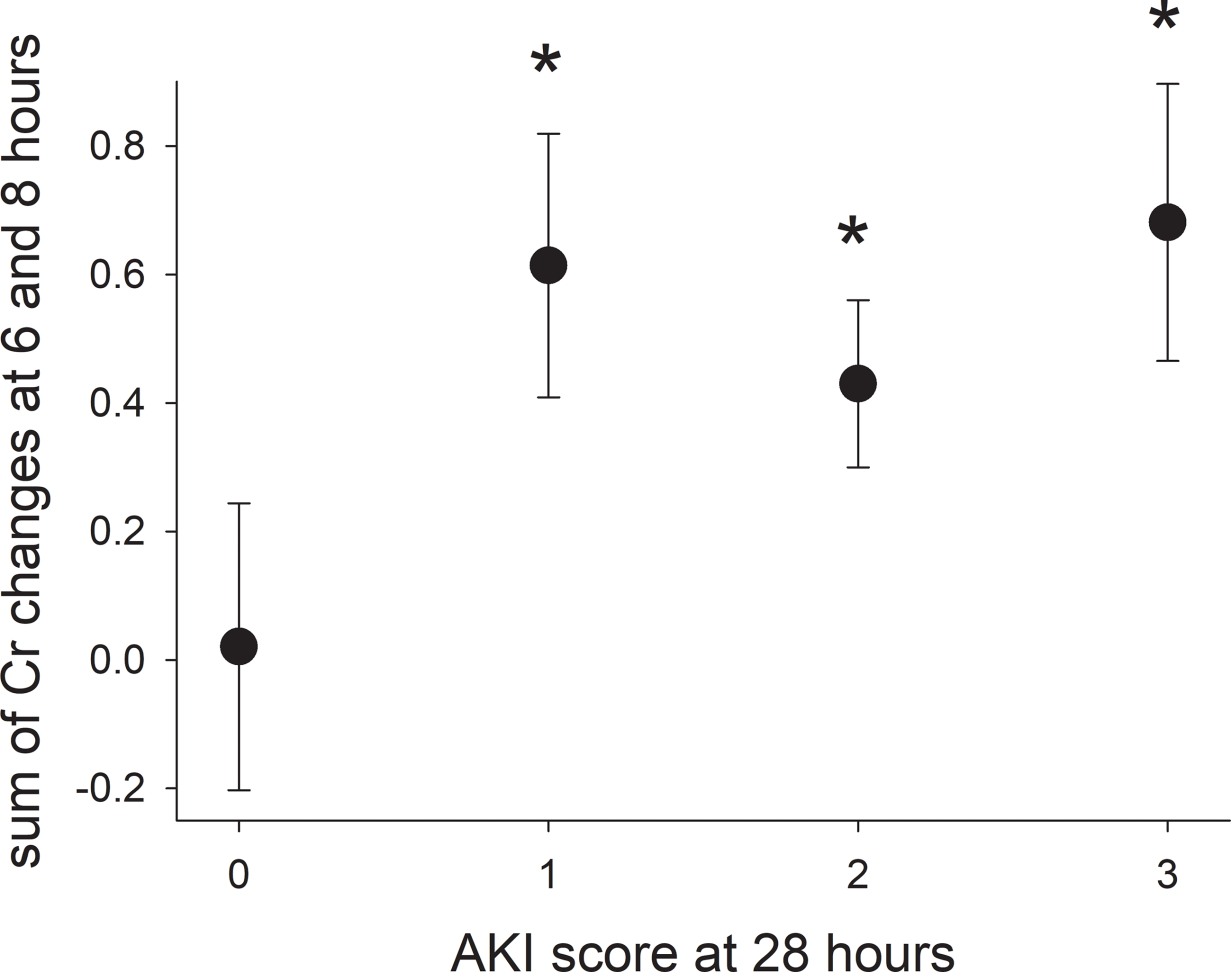
The sum of the increases in serum creatinine concentration at hours 6 and 8 (2 and 4 hours after ending the infusion) for pigs with varying severity of acute kidney injury. Asterisks indicate a significant difference (*p*-value < 0.05) compared with pigs without acute kidney injury (score of 0).

Increases in creatinine concentration at 6, 8, and 10 hours relative to 4 hours were not predictive of whether or not a pig would have AKI at 28 hours. However, the sum of creatine concentration increases at 6 and 8 hours from 4 hours was significantly higher for pigs with AKI at 28 hours (**Figure 4**), but did not identify pigs that would develop mild, moderate, or severe AKI. Including the increase in creatinine concentration at 10 hours did not improve predictability and, from a clinical perspective, would delay identifying pigs at risk of developing AKI that would benefit from early intervention to minimize renal damage and systemic toxicity.

### 3.3 Serum methotrexate concentrations

**Figure 5:**
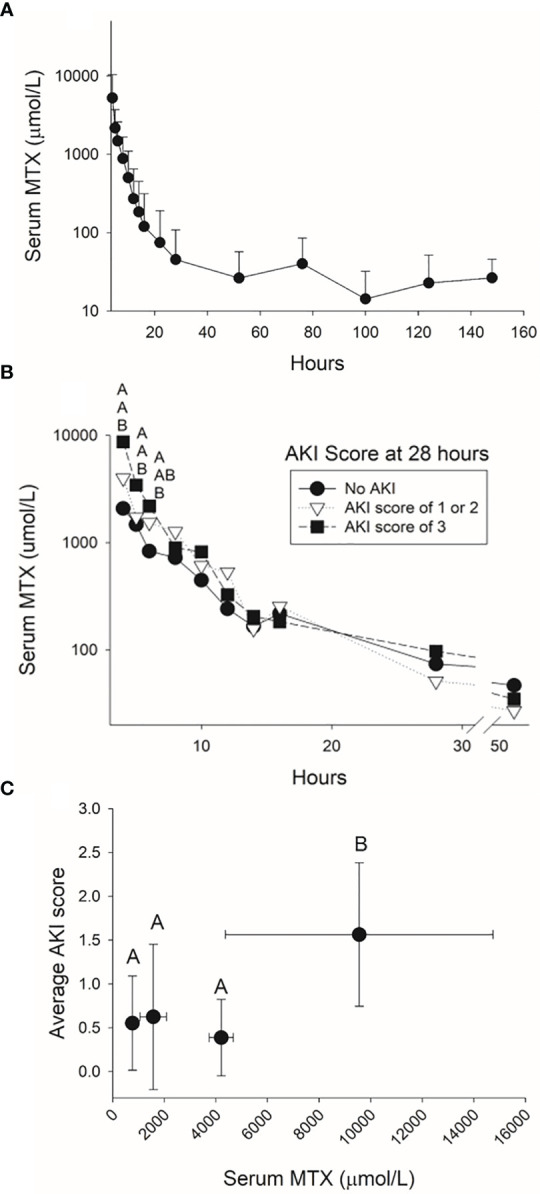
Serum methotrexate concentrations measured in the pig for 144 hours after the infusion of 4 g/kg of methotrexate **(A)** and for the first 48 hours after the infusion for pigs with varying severities of acute kidney injury at 28 hours **(B)**. The average acute kidney injury score based on the increases in serum creatinine concentration during the 24 hours after completing the 4-hour methotrexate infusion **(C)** was higher when serum concentrations at the end of the infusion were higher than 5,000 µmol/L.

There was rapid decline in serum MTX concentration during the first 24 hours after the infusion followed by a slower decline thereafter (**Figure 5A**), which is consistent with published two-compartment and three-compartment models of MTX elimination. At 52 hours, the mean serum MTX concentration was 28 μmol/L (± 27 μmol/L) and 81% of the pigs had serum MTX concentrations higher than the 1 μM concentration considered as evidence of delayed clearance among children receiving HDMTX. At 76 hours after the start of the HDMTX infusion, 18 (69%) out of 26 pigs had a serum MTX concentration > 0.1 μM, which is comparable to the 17 (61%) that had an elevated serum creatinine concentration indicative of AKI.

Pigs that developed mild, moderate, or severe AKI at 28 hours had significantly higher serum methotrexate concentrations at conclusion of the infusion (hour 4) than pigs without AKI (**Figure 5B**). For this model of HDMTX renal toxicity, serum MTX concentrations greater than 5,000 µM at conclusion of the infusion resulted in a higher AKI score averaged from the first 10 measurements of creatinine concentration after the infusion (**Figure 5C**).

**Figure 6:**
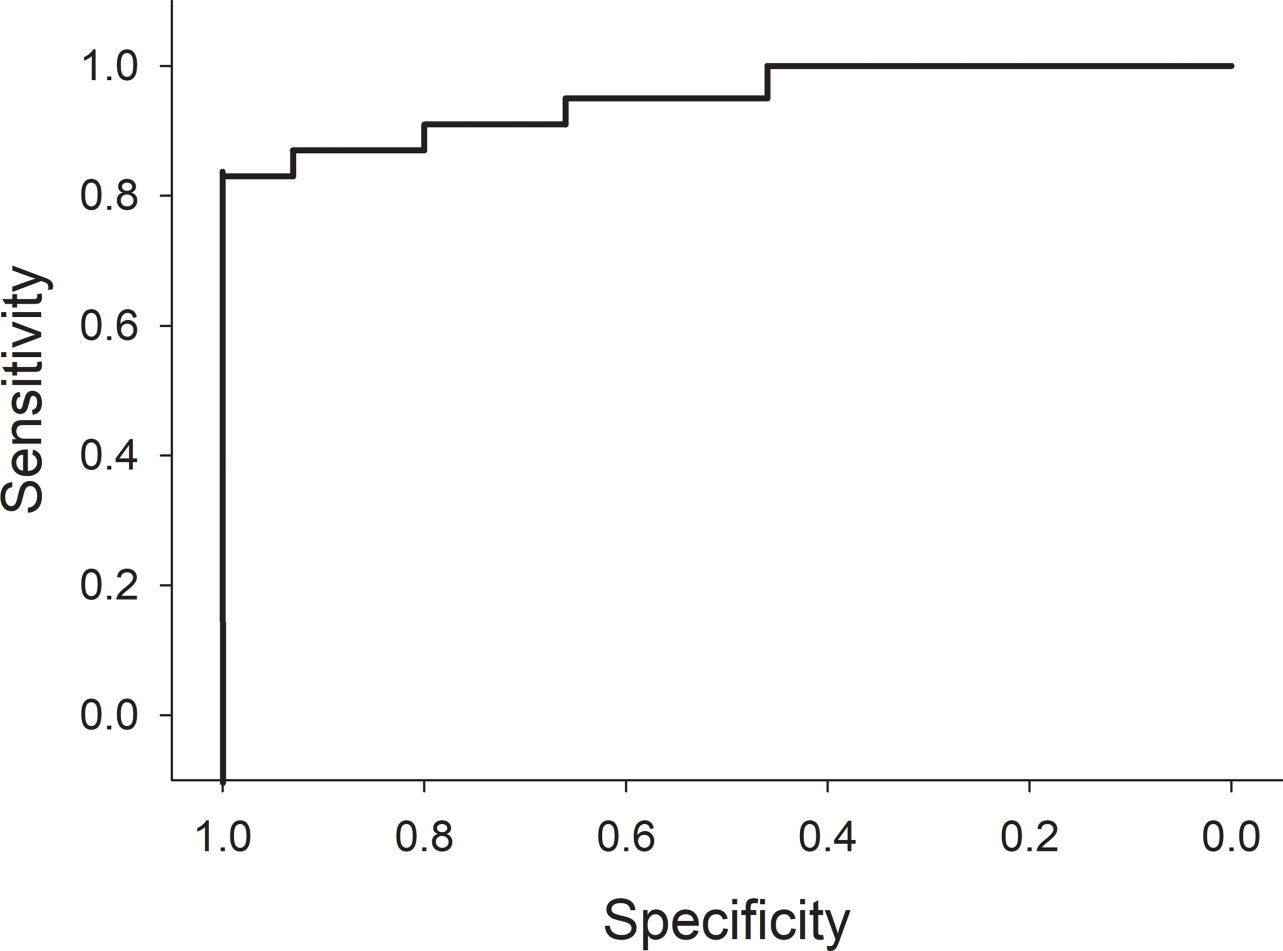
Receiver operator curve using a decrease of 90% or less in serum methotrexate levels within 4 hours from the time of completion of the 4-hour methotrexate infusion to predict acute kidney injury at 24 hours.

The rate of MTX elimination during the initial 4 hours after the end of the infusion was associated with the presence of AKI at 24 hours. Specifically, pigs with serum MTX concentrations at hour 8 that were greater than 10% of the serum MTX concentration at conclusion of the infusion were at greater risk of AKI (**Figure 6**; *p* = 0.03; *n* = 38; OR 0.46; 95% CI 0.22–0.94). In line with this, pigs with severe AKI at 28 hours had cleared less MTX at hour 8. The percentage decreases of MTX concentration at hours 10 and 16 hours were less predictive (*p*-values of 0.14 and 0.23, respectively). Among the pigs given a second dose of MTX, the rate of decrease in serum MTX concentration during the 4 hours after the second infusion was slower in pigs with AKI at the time of dosing than in pigs without AKI, particularly when compared with pigs that did not develop AKI after the second dose.

## 4 Discussion

Renal clearance is the major route for MTX elimination and the early identification of impaired MTX clearance is essential for beginning interventions to prevent the further deterioration of renal functions. It has been suggested that intrarenal precipitation of MTX is the leading pathological mechanism responsible for AKI in patients treated with high-dose MTX, especially when a very high MTX dose is infused over a few hours, as it is in patients treated for osteosarcomas. Hyperhydration and urine alkalinization are used as supportive care to prevent the formation of MTX crystals. However, the degree of alkalinization together with hydration volume and duration differs between cancer treatment protocols (30, 31). Some protocols increase the hydration volume in case of AKI, but it has never been confirmed if this prevents further deterioration of renal functions. It is also common to administer extra bicarbonate to patients with low urine pH during MTX infusion and it has even been suggested urine alkalinization alone can prevent MTX-induced AKI (32).

This study infused weaned pigs over 4 hours with a high volume (i.e., 250 mL/m^2^/h) of high-dose MTX (4 g/kg of MTX; equivalent to 80 g/m²) as a translational model for studying MTX-induced AKI. The 4 g/kg of MTX exceeded the 1–12 g/m² used in current cancer treatment protocols for leukemia and osteosarcoma, but it is similar to the 88 g/m^2^ administered to children with cancer (33, 34) and was infused without urine alkalinization to increase the risk of AKI. This approach caused AKI in 76% of the pigs within 28 hours after the start of the HDMTX infusion. The presence of MTX crystals in the kidneys of pigs that were euthanized before 52 hours suggests that this animal model is relevant to AKI in patients caused by infusions of HDMTX. Interestingly, no pigs had MTX crystals in their kidneys at autopsy 6 days after the HDMTX infusions. The high incidence of HDMTX-induced AKI among the pigs exceeds the rate of 2%–12% found in patients (35). The increases in serum creatinine concentration measured in the pigs mimic clinical data (36) and the 0.3 to 0.5 mg/dL increase for pigs with mild AKI is similar to the 1.5-fold increase for humans considered to have delayed elimination induced by HDMTX (37).

Different clinical parameters are considered to influence the risk of MTX-induced AKI. These include age, sex, genetics, serum total protein at the time of dosing, concomitant drugs, previous or subclinical episodes of AKI, and other unknown contributing factors (38–40). Variation among patients for these parameters confound clinical studies of MTX renal toxicity and complicate interpretations. These variables were minimized by using juvenile pigs of a similar age and body weight, and of the same genetic lineage that were given identical care and nutrition, and by not co-administering other chemotherapeutic agents. Still, there was variation among pigs in serum MTX concentrations, increases in serum creatinine concentration, and presence and severity of AKI. This replicates the unexplained variation in serum MTX concentrations and incidences and in severities of systemic toxicity and AKI among patients treated with HDMTX (10, 38), including pediatric patients treated with 33.6 g/m^2^ (33). The pig model provides an opportunity to identify factors that contribute to AKI risk and evaluate the efficacy of interventions.

### 4.1 Serum creatinine and AKI

The recovery of nearly 50% of the pigs from AKI mimics the potential of patients to regain normal renal clearance after MTX-induced injury (41, 42). However, only 4 of the 21 pigs (19%) that had moderate or severe AKI at or before 28 hours recovered before 148 hours. Of concern is that nephrons have a limited capacity for recovery and tubular dysfunction may persist for months (8). Any permanent loss of nephrons during HDMTX therapy depletes renal reserve capacity and increases the risk of AKI in subsequent HDMTX infusions and the future risk of chronic kidney disease. An unresolved question is what components of the nephron are capable of repair and recovery of functions. A strength of the pig model is the ability to address these questions and the need to understand the impact of HDMTX therapy during childhood on renal functions later in life (43). Of interest is how the cumulative increase in creatinine concentration during the initial hours after completion of the infusion may be an early determinant of AKI.

### 4.2 Serum MTX and AKI

The 4,751 µmol/L (+ 4,764 µmol/L) average MTX serum concentration at the end of the 4-hour HDMTX infusion exceeds the > 700 µM considered necessary for treating osteosarcoma (44). Only three pigs did not achieve this concentration at conclusion of the infusion. Methotrexate concentrations exceeded 1,500 µM at conclusion of the infusion for 69% of the pigs and five pigs had a serum MTX concentration greater than 10,000 µM. The wide variation in serum MTX concentrations and clearance among the pigs mimics the several-fold range of serum MTX concentrations and variable pharmacokinetics among pediatric patients [ (6, 45–47) (6, 46–48)]. The high MTX concentrations, especially without hyperhydration and urine alkalinization, increase the risk of toxicity and impaired renal clearance, and (10) certainly contributed to the high incidence of AKI among the pigs.

There was a clear association between serum MTX concentration at the end of infusion and risk of AKI within the first 28 hours after start of the HDMTX infusion. Concentrations of MTX at the end of the infusion that exceeded 5,000 µmol/L corresponded with higher AKI scores, particularly after hour 14. This highlights the importance of monitoring MTX steady-state concentrations during the infusion to adjust dosing, and, thereby, reduce toxicity and the incidence and severity of AKI (46). Of the seven pigs with MTX concentrations less than 1,000 µmol/L at the end of the infusion, four developed transient mild-to-moderate AKI during the first 28 hours, with one pig continuing to have moderate AKI at hours 28 and 52.

The majority of MTX is eliminated by glomerular filtration and renal tubular secretion (48, 49). Although glomerular filtration is responsible for the initial exponential decline in serum MTX concentration, tubular secretion is a saturable pathway with an increasing role in the elimination of MTX at lower concentrations (37). A novel finding from the dense sampling of the pigs after the infusion is that a slower exponential decline in serum MTX concentration during the first 4 hours after the infusion is a sensitive and early indicator of impaired renal clearance and increased risk of AKI. This has not been previously recognized because pharmacokinetic modeling of MTX clearance has relied largely on samples collected from patients at or after 24 hours to identify patients with delayed elimination and renal damage (50). This approach has had limited success predicting impending renal injury, impaired clearance, and systemic toxicity (45, 51, 52). Samples collected 6 hours after ending the infusion (44) may still not be early enough to characterize the steep exponential decline in serum MTX concentration that occurs during the initial hours after the infusion (44, 53). Our findings with the pig model indicate that the initial exponential decline in MTX concentration can be used in monitoring of serum MTX concentration during the infusion for earlier identification of patients with delayed elimination and renal damage.

The half-life of serum MTX during the first 24 hours after dosing has been estimated at about 150 min (42). In the present study, the hour 5 serum MTX concentrations averaged 60% (±30%) of those measured at the end of administration (hour 4) and at hour 6 were 37% (±20%), implying that the half-life during the initial 2 hours after the infusion is shorter than 2 hours. The shorter half-life may reflect the high initial concentrations (33). The reduction in MTX clearance, hence longer half-life, and increase in serum creatinine during the first 4 hours after the infusion is consistent with MTX crystals obstructing nephron tubules and decreasing glomerular filtration without an apparent relationship with renal blood flow (54).

When sufficient numbers of nephrons are compromised and filtration capacity is diminished, the ability to rapidly clear MTX, creatinine, and other filtered molecules declines (55). This prolongs the exposure of the renal tubular epithelium to toxic concentrations of MTX, which can compromise tubule transport and the elimination of toxins and other drugs (56). The strategy of hyperhydration and urine alkalinization increases MTX solubility in urine, but it does not prevent the formation of MTX crystals in the nephrons (57), even without high serum concentrations (11), nor does it enhance filtration by remaining glomeruli. Although high-dose leucovorin rescue addresses the systemic toxicity for patients with MTX nephrotoxicity and reduced clearance, there is little or no evidence that this intervention reduces MTX concentrations in the nephrons (58). The pig model can be used to evaluate interventions that preserve glomerular filtration and tubular secretion, minimize renal and systemic damage, and will enhance renal recovery from current and future therapy regimens.

The presence of MTX in the feces of the pigs corroborates the presence of hepatic clearance associated with biliary secretion (59). This alternative pathway of elimination is responsible for 10%–30% of MTX elimination and is mediated by transporters in the intestine and liver (60, 61). Hepatotoxicity associated with HDMTX (62) has the potential to limit this pathway of elimination.

### 4.3 Identifying patients at risk of MTX-induced renal injury—a new paradigm

**Figure 7:**
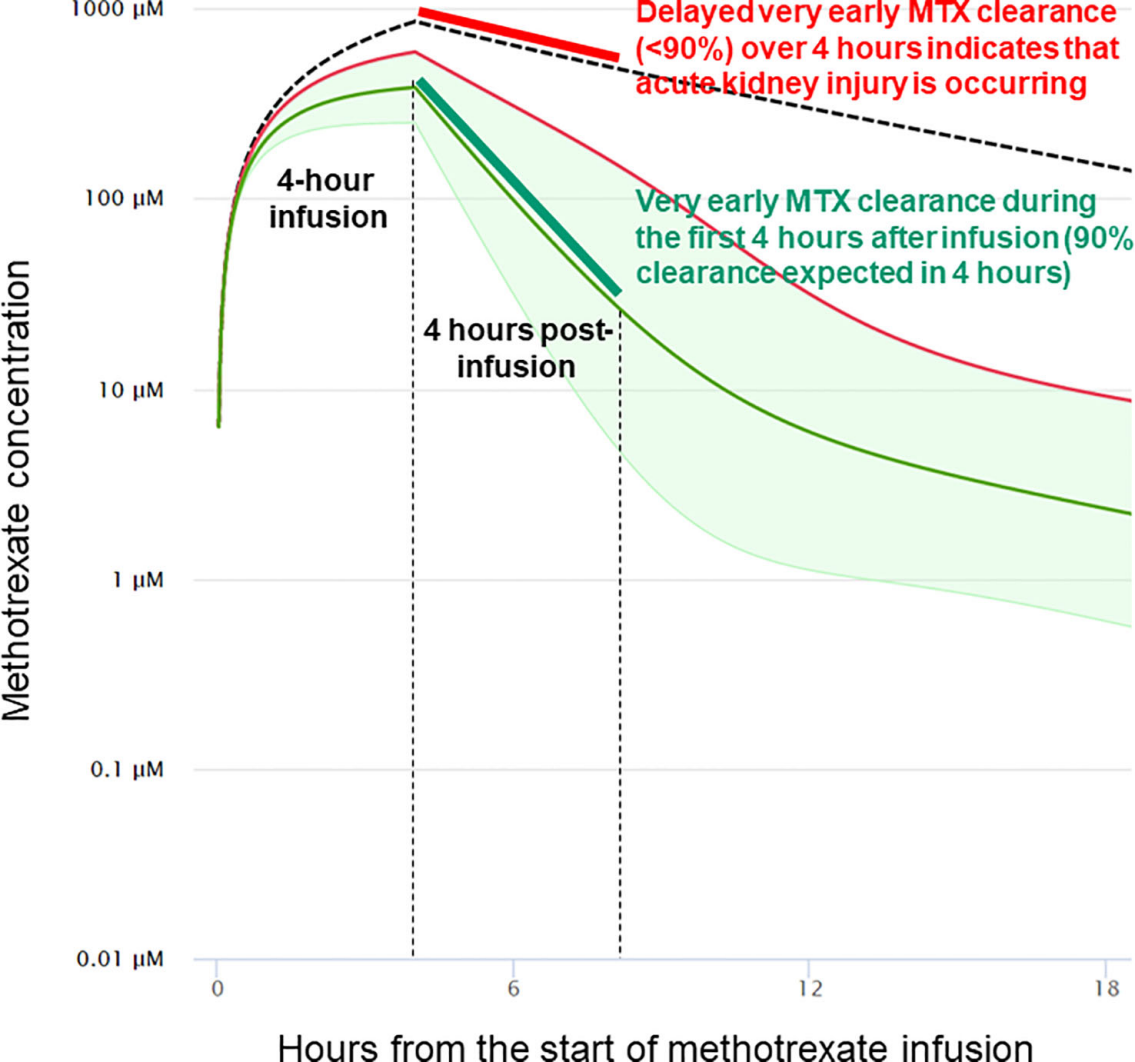
Impact of very early clearance of methotrexate on the probability of delayed elimination and acute kidney injury. In this example from models validated in humans, the 4-hour methotrexate clearance analyzed in the pig model is estimated to accurately predict methotrexate clearance and acute kidney injury at 24 and 48 hours from the start of the methotrexate infusion. The model used is taken from Resonance Study Manager (ResonanceHealth.org), which uses the validated model at MTXPK.org.

Identifying factors that increase the risk of HDMTX-induced renal damage is of paramount importance. The variations in serum MTX concentration and AKI, despite the minimization of known contributing variables in this experimental model, highlights the need for new biomarkers of delayed elimination of MTX and development of AKI. In this regard, the rate of decline in MTX concentrations during the first 4 hours after completion of the infusion (hours 4 through 8 in this model) was highly predictive of development of AKI, with a receiver operator curve that has large areas with either perfect sensitivity or perfect specificity (**Figure 6**). The half-life of MTX in juvenile pigs is approximately 1.3 hours, so the elimination of 90% of MTX would be expected in 4 hours (slightly more than three half-lives). Indeed, all animals with lower rates of MTX elimination than 90% during the first 4 hours after stopping the infusion developed AKI. This leads to a new paradigm for early detection of delayed MTX elimination that is (1) based on very early measurements when renal protective measures, such as increased hyperhydration, more aggressive urine alkalinization and glucarpidase will be most beneficial; (2) based on the rate of MTX elimination rather than any single level taken at a single time point, which serves as a surrogate marker of decreased glomerular filtration rate (GFR), which can be measured in real time without waiting for the rise in creatinine concentration, which will inevitably occur 1–2 days after renal injury; and (3) independent of the MTX dose, duration of infusion, and patient characteristics, as the rate of decrease of MTX concentration during the first 4 hours reflects GFR during those 4 hours and obviates the need for complex nomograms and single-time-point thresholds that differ based on MTX dose, MTX infusion duration, time from the start of MTX infusion, patient sex, and serum creatinine concentration (**Figure 7**).

Renal clearance varies among individual humans and pigs owing to differences in numbers of functional nephrons (63) that can originate during nephrogenesis (64, 65). Corresponding with this, the normal decline in nephron numbers that occurs with aging (66) coincides with the greater risk in adults than in children of delayed MTX clearance and higher peak serum creatinine concentration, even at lower MTX doses (67). There is growing interest in pharmacogenomics for identifying genes and gene products that contribute to MTX clearance (53, 68). These efforts combined with metabolomic analysis of urine samples at the start and conclusion of MTX therapy (16) may reveal genetic factors that are predictive of risks.

## 5 Conclusions

Weaned juvenile pigs given 4-hour HDMTX infusions represent a valuable preclinical model for MTX-induced AKI and can be used to study biological, environmental, and iatrogenic factors that increase the risk of AKI. This includes administration of pantoprazole (69), cisplatin, and other conventional chemotherapeutic agents that are nephrotoxic (70, 71) and could increase the incidence and severity of AKI caused by HDMTX. Other drug–HDMTX combinations can be evaluated as potential risk factors for increased incidence and severity of AKI in pediatric patients (72, 73). This preclinical animal model can potentially be used to evaluate how the current supportive care with hyperhydration and urine alkalization can be optimized. Furthermore, the model can be used to identify new biomarkers and protocols for early identification of reduced renal clearance and to study the underlying pathophysiological mechanisms of MTX-induced AKI. Once validated in humans through measurement of MTX at two early time points (0 and 4 hours after completing the infusion), the rate of MTX elimination during this time should be the only biomarker needed for very early detection of potential AKI. The model needs to be extended to include responses to 24-hour infusion. Early mitigation using increased hyperhydration, alkalinization, and glucarpidase will prevent extensive and permanent renal dysfunction, reduce the systemic MTX toxicity, and lower the costs associated with delaying treatment (69).

## Data availability statement

The original contributions presented in the study are included in the article/supplementary material. Further inquiries can be directed to the corresponding author.

## Ethics statement

The animal study was reviewed and approved by the University of Memphis Institutional Animal Care and Use Committee.

## Author contributions

Conceptualization and funding acquisition: RB and SH. Methodology: RB, SH, and TW. Conduct of study phases using animals: RB, KB, and TW. Sample analysis: RB and TW. Data analysis and interpretation: RB, SH, TM, and XC. Preparation of the manuscript: RB, SH, and TM. All authors contributed to the article and approved the submitted version.
